# Halide Chemistry in Tin Perovskite Optoelectronics: Bottlenecks and Opportunities

**DOI:** 10.1002/anie.202213966

**Published:** 2022-12-21

**Authors:** Luis Lanzetta, Thomas Webb, Jose Manuel Marin‐Beloqui, Thomas J. Macdonald, Saif A. Haque

**Affiliations:** ^1^ Department of Chemistry and Centre for Processable Electronics Molecular Sciences Research Hub Imperial College London London W12 0BZ UK; ^2^ Physical Science and Engineering Division KAUST Solar Center (KSC) King Abdullah University of Science and Technology (KAUST) Thuwal 23955-6900 Saudi Arabia; ^3^ Department of Physical Chemistry University of Málaga Andalucia-Tech Campus de Teatinos s/n 29071 Málaga Spain; ^4^ School of Engineering and Materials Science Queen Mary University of London London E1 4NS UK

**Keywords:** Halide Chemistry, Optoelectronics, Solar Cells, Stability, Tin Halide Perovskites

## Abstract

Tin halide perovskites (Sn HaPs) are the top lead‐free choice for perovskite optoelectronics, but the oxidation of perovskite Sn^2+^ to Sn^4+^ remains a key challenge. However, the role of inconspicuous chemical processes remains underexplored. Specifically, the halide component in Sn HaPs (typically iodide) has been shown to play a key role in dictating device performance and stability due to its high reactivity. Here we describe the impact of native halide chemistry on Sn HaPs. Specifically, molecular halogen formation in Sn HaPs and its influence on degradation is reviewed, emphasising the benefits of iodide substitution for improving stability. Next, the ecological impact of halide products of Sn HaP degradation and its mitigation are considered. The development of visible Sn HaP emitters via halide tuning is also summarised. Lastly, halide defect management and interfacial engineering for Sn HaP devices are discussed. These insights will inspire efficient and robust Sn HaP optoelectronics.

## Graphical abstract

Efforts to boost tin halide perovskite optoelectronics have focused heavily on the instability of Sn^II^, while other inconspicuous chemical processes have remained unexplored. This Minireview highlights the key role of native halide chemistry in the stability, device performance, ecological impact and prospects for visible light emission in this class of materials. Halide management routes towards viable optoelectronics are also proposed.

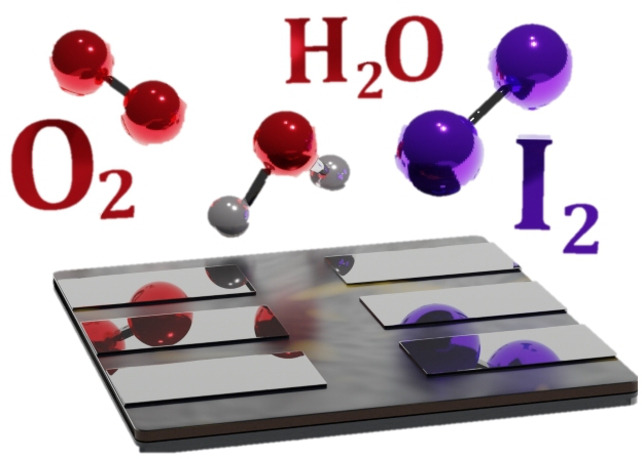

## 1 Introduction

Lead halide perovskites (Pb HaPs) are attracting attention for their outstanding semiconducting properties, i.e. tuneable band gaps, defect tolerance and solution processability.[^1^, ^2^] This has made Pb HaPs top candidates towards next‐generation optoelectronics, with solar cell efficiencies nearing 26 %, external quantum efficiencies in light‐emitting diodes (LEDs) over 28 % and ultralow detection limits in X‐ray scintillators.[^3^, ^4^, ^5^] However, Pb toxicity raises concerns on their environmental and health effects. For example, upon exposure to environment (e.g. via device encapsulation failures), Pb HaPs may release Pb^2+^ into the soil by forming degradation products that are slightly soluble in water (e.g. PbI_2_; solubility product constant: ≈10^−8^).^[6]^ Alternatively, tin halide perovskites (Sn HaPs) have emerged as the leading Pb‐free candidate, providing excellent optical and charge transport properties and decomposing into virtually inert Sn‐based products with lower bioavailability (i.e. SnO_2_, solubility product constant: ≈10^−64^).^[7]^

However, Sn HaP optoelectronics have not yet matched the potential of their Pb counterparts. The Sn^2+^ species in the perovskite is highly unstable and its conversion to Sn^4+^‐based chemical products may occur at any stage of the life cycle of the material, even in presence of trace amounts of oxidants (e.g. atmospheric O_2_).^[8]^ Sn HaPs also exhibit p‐type doping that leads to short carrier lifetimes and negatively impacts device performance.^[9]^ Degradation under ambient environmental conditions can also p‐dope Sn HaPs, where O_2_ may act as an electron scavenger [Eq. 1].^[2]^

(1) $$\mathrm{Sn}{}^{2+}+O{}_{2}\to \mathrm{SnO}{}_{2}+2\;h{}^{+}$$

Intrinsic Sn^2+^ vacancies (V_Sn_) and bulk Sn^4+^ states have also been identified as sources of p‐doping as per the following processes [Eq. 2, 3].^[10]^

(2) $$V{}_{\mathrm{Sn}}\to V{}_{\mathrm{Sn}}{}^{2-}+2\;h{}^{+}$$

(3) $$\mathrm{Sn}{}^{4+}\to \mathrm{Sn}{}^{2+}+2\;h{}^{+}$$

Lastly, Sn HaPs suffer from faster crystallisation vs Pb HaPs during typical solution deposition methods, which gives rise to morphological defects and suboptimal device quality (e.g. shunting).^[11]^

To date, research efforts have focused heavily on harnessing Sn chemistry to tackle the issues summarised above. Examples include V_Sn_ filling with vacancy modulation additives (e.g. SnF_2_),^[12]^ eliminating Sn^4+^ from solutions using reducing agents^[13]^ and exploiting the coordination between Lewis bases and Sn^2+^ to control growth and increase stability.^[14]^ While these approaches have driven Sn HaPs optoelectronics forward, further progress requires full understanding of inconspicuous chemical processes beyond Sn‐related phenomena. In particular, the halide chemistry in Sn HaPs has been largely underexplored despite its key role in their final properties. Indeed, Sn HaP stability is severely compromised by the formation of iodine (I_2_) in iodide (I^−^) based Sn HaPs, following degradation.[^8^, ^15^] Oxidative stress from native I_2_ may also contribute to unwanted p‐doping in Sn HaPs via the proposed process [Eq. 4].[^2^, ^8^]

(4) $$\mathrm{Sn}{}^{2+}+2\;I{}_{2}\to \mathrm{SnI}{}_{4}+2\;h{}^{+}$$

Alongside V_Sn_ and Sn^4+^, iodine interstitials (I_i_) in Sn HaPs are electron acceptor defects that may equally account for the p‐type behaviour in these materials [Eq. 5].^[2]^

(5) $$I{}_{i}\to I{}_{i}{}^{-}+h{}^{+}$$

The tuneable Lewis basicity of halides in Sn HaPs (from softer to harder: I^−^<Br^−^<Cl^−^<F^−^) may also be useful to promote homogeneous growth via the formation of Sn^2+^ halide adducts, and even to selectively sequestrate Sn^4+^ in solution.^[16]^ Altogether, these examples illustrate the importance of halide engineering towards Sn HaPs with high performance and stability. This can address the limitations of Sn HaPs, but also extend their functionality, e.g. towards visible light emitters.

This Minireview highlights the challenges and research opportunities arising from halide chemistry in Sn HaPs, focusing on its link to i) material stability and device performance, ii) the ecological impact of this class of materials and iii) their visible light emission. Potential directions for effective halide defect management and interfacial engineering in Sn HaPs devices are also proposed.

## 2 Impact of Halides on Stability and Performance

**Figure 1:**
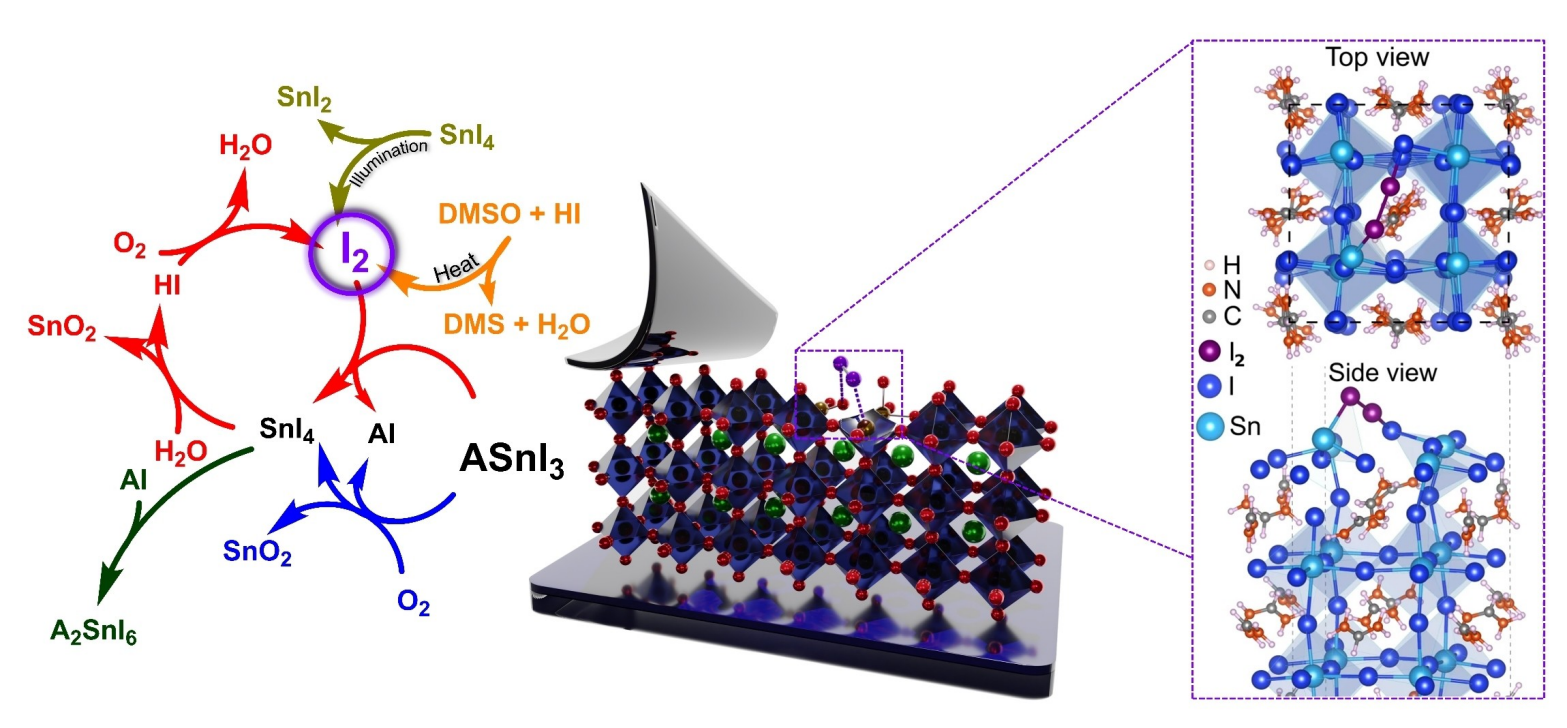
Left: chemical pathways leading to I_2_ formation in Sn HaPs, i.e. photoreduction of SnI_4_ (yellow),^[21]^ decomposition of DMSO to dimethyl sulfide (DMS, orange),^[15]^ O_2_‐ and H_2_O‐driven degradation of Sn HaP (blue and red).^[8]^ Right: degradative interaction of I_2_ with the (001) FASnI_3_ surface simulated via first principles calculations. Reproduced with permission from ref. [8].

The formation of I_2_ within iodide‐based perovskite structures is becoming increasingly recognised. In Pb HaPs, I_2_ has been shown to arise from numerous processes such as the photodecomposition of PbI_2_ ^[17]^ and the combination of I_i_^+^/I_i_^−^ pairs under illumination.^[18]^ In contrast, very few works have investigated the evolution and role of I_2_ within Sn HaPs. The following section describes the chemistry of I_2_ formation in Sn HaPs (summarised schematically in Figure 1) and its relevance to stability and solar cell performance. These findings clearly point to the importance of halide substitution as a potential route to more stable Pb‐free perovskite optoelectronics.

### 2.1 Formation of I_2_ in Sn HaPs

Saidaminov et al. observed that dimethyl sulfoxide (DMSO), a commonly employed solvent in perovskite precursor solutions, can oxidise SnI_2_ to SnI_4_ upon heating.^[19]^ A detailed investigation by Pascual et al. identified that native hydroiodic acid (HI) can promote the DMSO‐mediated degradation of SnI_2_ to SnI_4_, proposing I_2_ formation in this process as the reason behind the oxidation of Sn^2+^ in solution.^[15]^

Recently, we explored Sn HaP film decomposition under ambient environmental conditions (O_2_+humidity) and detected SnI_4_ as a result of an O_2_‐driven degradation process,^[8]^ in agreement with previous reports.^[20]^ A key feature of SnI_4_ is its rich chemical reactivity which can impact stability. Indeed, we showed that SnI_4_ can either i) combine with iodosalts (AI, where A is a monovalent cation) and generate a vacancy‐ordered double perovskite analogue (A_2_SnI_6_) or ii) more importantly, participate in a hydrolysis reaction with H_2_O to give HI, which in turn reacts with O_2_ to form I_2_. The halogen (I_2_) was then shown to further decompose perovskite (Figure 1, right) and produce more SnI_4_ in a cyclic degradation process. In essence, this mechanism causes the breakdown of perovskite triggered by moisture and O_2_, where native iodide ions in the lattice evolve to form aggressive I_2_ species.

Further pathways leading to I_2_ formation have also been identified by Sanchez‐Diaz et al. Specifically, they reported the presence of a light‐activated ligand‐to‐metal charge transfer (LMCT) process in SnI_4_ within the perovskite leading to the formation of I_2_ and SnI_2_.^[21]^

Altogether, the formation of I_2_ can occur through multiple chemical reactions and conditions (e.g. solution and solid‐state; inert atmosphere and ambient conditions; dark and under illumination).

### 2.2 Halide Substitution in Sn HaPs

Despite its key role in determining materials stability, the number of studies addressing the presence and effect of I_2_ as an oxidiser within Sn HaPs remains limited. As per Pb HaPs, it is likely that reducing the number of intrinsic point defects and improving the morphology of the film will improve the tolerance to ambient conditions and halogen formation.^[22]^ Another approach may be the in situ neutralisation of I_2_ through the addition of sacrificial reductants to Sn HaPs, such as NaBH_4_.^[21]^ While these strategies are helpful to improve material and device stability, only limiting the intrinsic ability of Sn HaP to form I_2_ is expected to address the root cause of halogen‐mediated degradation.

The substitution of X‐site I^−^ in the ASnX_3_ perovskite structure with bromide (Br^−^) is one approach to mitigate I_2_ formation in Sn PSCs. Based on the trend of halogen oxidation strength (F_2_>Cl_2_>Br_2_>I_2_), the removal of I^−^ by Br^−^ will most likely result in a weaker tendency of Sn HaP to form halogens. Similarly, Sn^4+^ compounds containing Br^−^ are less susceptible to undergo LMCT photoreduction reported for SnI_4_, thus limiting halogen evolution.^[23]^ In terms of lattice energy, the smaller ionic radii of Br^−^ lead to stronger Sn−X bonds relative to I^−^, further increasing the stability of the perovskite system. Due to their small size, the remaining halides (Cl^−^ and F^−^) do not easily incorporate into lattice X sites;^[24]^ however, the use of SnF_2_ or SnCl_2_ V_Sn_ filling additives to sequester Sn^4+^ in solution may be beneficial by limiting the presence of the species that can trigger halogen formation as per the mechanisms reviewed above (i.e. SnI_4_, HI; see Figure 1, left).

**Figure 2:**
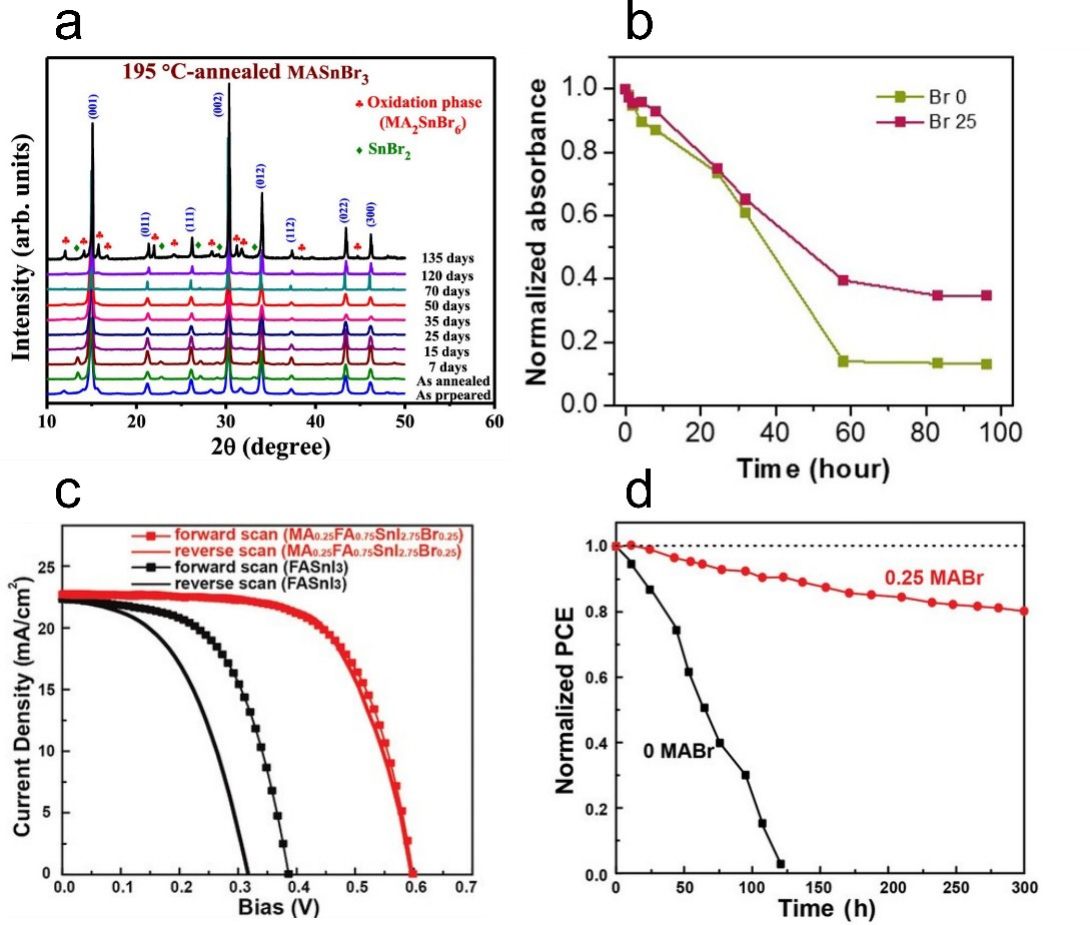
a) X‐ray diffraction patterns of MASnBr_3_ showing no changes in structure after 4 months of ambient aging. Reproduced with permission from ref. [25]. b) Optical degradation test of FASnI_3−*x*_Br_*x*_ showing higher ambient stability in mixed‐halide compositions (*x*=0.25). Reproduced with permission from ref. [26]. c) Current density–voltage curves of Sn HaP solar cells with varying Br^−^ content and d) their operational stability tests under AM1.5 light soaking. Reproduced with permission from ref. [31].

Sn HaPs prepared with high fractions of Br^−^ have been shown to exhibit high stability, with i) Ippili et al. demonstrating that MASnBr_3_ (where MA is methylammonium) can retain its crystal structure with minimal changes over 4 months in ambient conditions (Figure 2a)^[25]^ and ii) Lee et al. and Zhu et al. reporting higher optical stability after 100 h (Figure 2b) and lower free carrier densities in Br‐substituted FASnI_3_ (where FA is formamidinium) and MASnI_3_, respectively.[^26^, ^27^] This suggests that the evolution of Br_2_ does not readily occur in the same way as I_2_. In addition to the thermodynamically unfavourable oxidation of Br^−^ and the increased Sn−Br bond strength, the lack of Br_2_ most likely originates from a stronger hydrogen bonding between the A‐site ammonium/amidinium hydrogens and Br^−^ ions relative to I^−^.^[28]^ Furthermore, I^−^ replacement with smaller Br^−^ reduces the unit‐cell dimensions, producing a more compact lattice that can potentially block O_2_ and moisture ingress; these being key factors in the conversion of SnI_4_ to I_2_.

The benefits of incorporating small fractions of Br^−^/Cl^−^ can also be extended to the performance of fully functioning optoelectronic devices. It is noteworthy that the only two literature examples reporting Sn HaP solar cell efficiencies exceeding 14 % include small fractions of Br^−^ in the absorber layer.[^29^, ^30^] The inclusion of Br^−^ has been shown to deactivate non‐radiative carrier recombination and improve the film microstructure. However, only a few reports have assessed the role of halide substitution in the chemical and operational stability of Sn HaP materials and devices. Lee et al. demonstrated that substituting 25 % of I^−^ by Br^−^ in FASnI_3_ not only improves device performance but also leads to i) a decrease of three orders of magnitude in free hole concentration and ii) better stability of encapsulated solar cells (84 % of initial efficiency kept after 1000 h under AM1.5 G light exposure).^[26]^ Similarly, Yu et al. reported solar cells based on MA_0.25_FA_0.75_SnI_2.75_Br_0.25_ with efficiencies of 9.31 % in contrast to 5.02 % in FASnI_3_ devices (Figure 2c). Devices with 25 % Br^−^ retained 80 % of their performance after 300 h, outliving I^−^‐based devices (Figure 2d).^[31]^ These improvements in stability have been attributed to an increase in V_Sn_ formation energy and higher crystal orientation upon Br^−^ incorporation. However, we hypothesise that limiting the I^−^ content in Sn HaPs may also mitigate halogen formation and p‐type doping pathways [Figure 1 (left) and Eq. (4) and (5), respectively]. In line with this, Khan et al. proposed that the addition of Br^−^ to Sn HaPs increases the formation energy of iodide vacancies (V_I_), limiting harmful HI generation and accounting for the lower Sn^4+^ content in Br^−^‐substituted films.^[32]^

The addition of Br^−^ leads to a trade‐off between light harvesting capability and intrinsic materials stability.^[33]^ Therefore, the need to retain an ideal band gap (for solar cell devices) limits the fraction of native I^−^ that can be replaced by Br^−^. However, opportunities for high Br^−^ fraction, wide band gap perovskites may emerge in tandem solar cells,^[34]^ transistors^[27]^ and visible light emitters (see Section 4).[^35^, ^36^]

## 3 Ecological Impact

**Figure 3:**
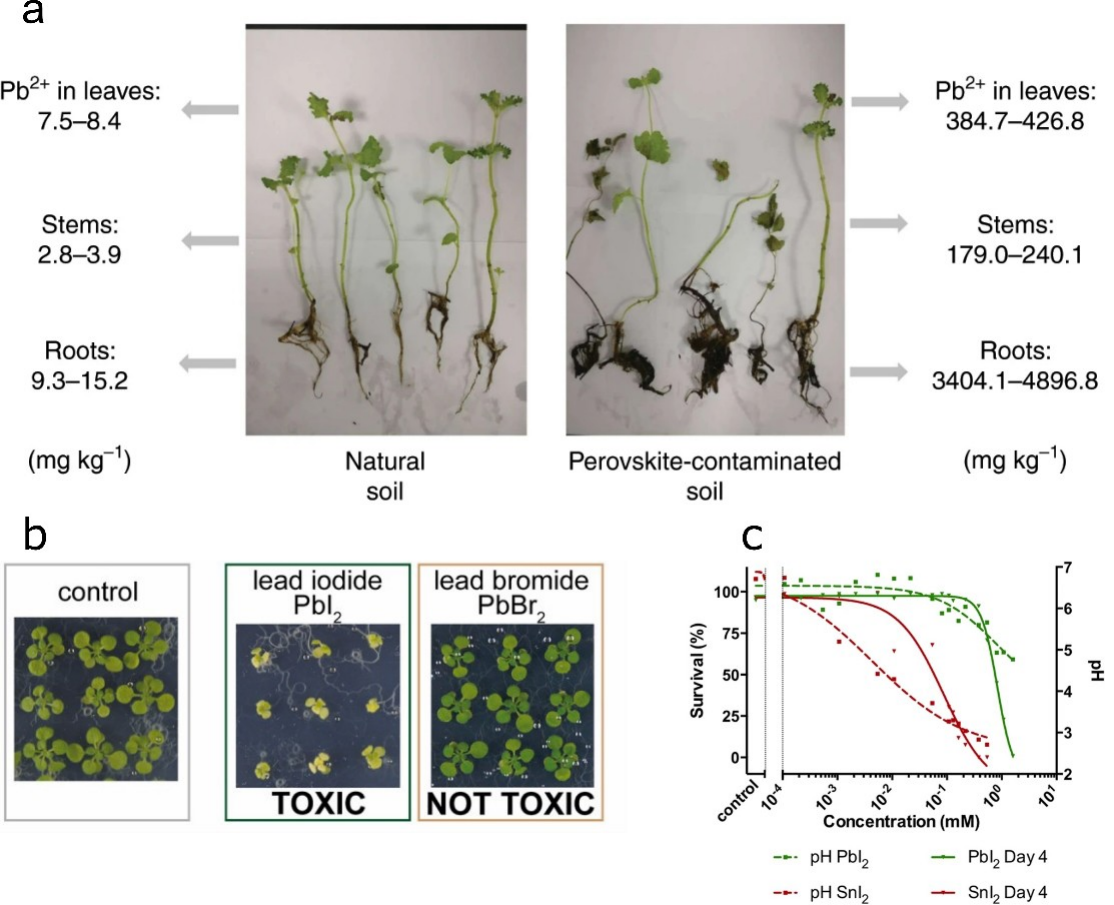
a) Pb uptake values of mint plants grown in natural soil (left) and in perovskite‐contaminated soil (right, 250 mg kg^−1^ MAPbI_3_). Reproduced with permission from ref. [37]. b) Toxicity effect of I^−^ vs Br^−^ ions from perovskite precursors in *Arabidopsis* plants. Reproduced with permission from ref. [38]. c) Correlation between zebrafish embryo survival and pH in PbI_2_/SnI_2_‐contaminated aqueous medium. Reproduced with permission from ref. [40].

Monitoring metal uptake by plants provides a simple way to assess the biological impact of HaPs. Li et al. evaluated Pb and Sn capture by growing mint on soil contaminated with MAPbI_3_/MASnI_3_ precursors.^[37]^ Upon adding 5 mg kg^−1^ of Pb/Sn HaP to soil, mint roots show a Pb uptake coefficient (defined as the metal content increase in plants relative to contaminant concentration added to soil) of ≈300 % (effects in plants shown in Figure 3a), while for Sn this is ≈40 %. These results highlight the lower bioavailability of Sn compared to Pb, suggesting that Sn HaPs may offer a safer technology with lower bioaccumulation.

To assess the toxicity of HaPs holistically, it is important to consider the effect of all constituting ions as well as the interplay between them. In the previous study by Li et al., the presence of MA^+^ was shown to exacerbate metal uptake, presumably by causing changes in soil pH.^[37]^ The contribution of halides in HaPs to plant toxicity was recently investigated by Hutter et al;^[38]^ MAPbI_3_ and its precursors were shown to induce smaller plants and lower chlorophyll production (Figure 3b) at much lower concentrations than Br^−^‐based precursors MABr and PbBr_2_ (5 vs 500 μM). While I^−^ itself is considered a micronutrient in plants,^[39]^ its chemical nature when in HaPs possibly accentuates the negative effects caused by the metal and the organic components. Further studies looking into the ecological impact of I^−^‐poorer compositions are pertinent to develop more eco‐friendly HaPs.

It is plausible that the above issues can occur in I^−^‐based Sn HaPs, although an additional concern in these materials is their potential acute toxicity to water organisms. Babayigit et al. analysed the effects of aqueous PbI_2_ and SnI_2_ on zebrafish embryos;^[40]^ while PbI_2_ led to embryonal malformations, SnI_2_ was observed to inhibit hatching and show higher lethality rates. This was attributed to the acidification of water due to SnI_2_ and SnI_4_ hydrolysis [Figure 3c; Eq. 6, 7].

(6) $$8\;\mathrm{SnI}{}_{2}+4\;H{}_{2}O+O{}_{2}\to 2\;\mathrm{SnI}{}_{4}+6\;\mathrm{Sn}\left(\mathrm{OH}\right)I+2\;\mathrm{HI}$$

(7) $$\mathrm{SnI}{}_{4}+2\;H{}_{2}O\to \mathrm{SnO}{}_{2}+4\;\mathrm{HI}$$

Hydrohalic acids [HI in Eq. (6) and (7)] thus represent the main safety challenge in Sn HaPs. To limit the ability of Sn HaPs and their degradation products to hydrolyse, it is therefore critical to increase their stability in water; water‐resistant coatings such as Al_2_O_3_ ^[41]^ and the synthesis of perovskites terminated with insoluble layers^[42]^ offer promising opportunities. Alternatively, the incorporation of metal‐sequestrating materials in devices such as chelating agents^[43]^ and cation‐exchange resins/minerals[^44^, ^45^] is a reasonable approach to limit the interaction of Sn halides with water.

## 4 Visible Sn HaP Emitters

Archetypal I^−^‐based Sn HaPs (e.g. MASnI_3_) exhibit near ideal band gaps for solar cells (≈1.30 eV). However, this limits their emission to the near‐infrared region.[^36^, ^46^] Substitution of I^−^ with Br^−^ provides an attractive approach to tune the emission properties of the perovskite. For example, Lai et al. showed that the partial incorporation of Br^−^ in MASnI_3_ gives LEDs emitting up to ≈667 nm (50 % Br^−^).^[36]^ Bandgap broadening upon halide substitution can be rationalised by i) stronger electron confinement in shorter metal−halide bonds leading to shallower conduction band minimum and ii) higher electronegativity in smaller halides causing deeper valence band maximum.^[47]^

### 4.1 Two‐Dimensional Sn HaP Emitters

**Figure 4:**
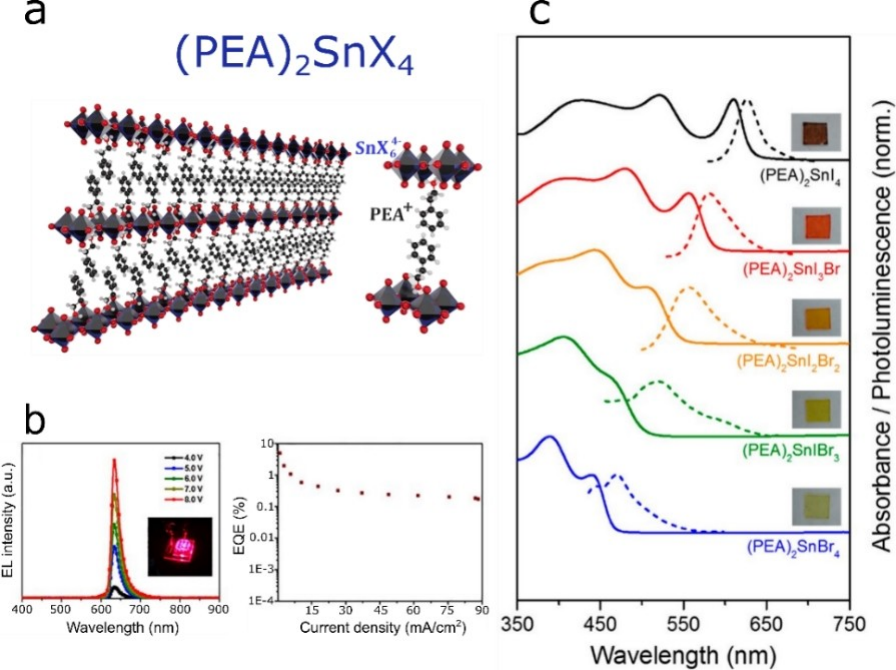
a) Structure of a 2D Sn HaP employing PEA^+^ as the organic spacer. b) Electroluminescence (left) and EQE of state‐of‐art (PEA)_2_SnI_4_ LEDs. Reproduced with permission from ref. [57]. c) Absorbance and PL of (PEA)_2_SnI_*x*_Br_4‐*x*_ visible emitters. Reproduced with permission from ref. [35].

Two‐dimensional (2D) Sn perovskites offer superior emission properties in the visible region (from ≈450 nm to ≈650 nm) as compared to their 3D counterparts.^[35]^ Layered perovskites incorporate bulky organic cations in the A‐site (Figure 4a), leading to higher stability and wider band gaps.^[48]^ Bandgap broadening arises from stronger carrier confinement,^[49]^ which in turn leads to exciton binding energies of over an order of magnitude higher in 2D perovskites relative to their 3D analogues.^[50]^ In our previous work, we demonstrated that transitioning from MASnI_3_ to (PEA)_2_SnI_4_ (PEA=phenylethylammonium) causes a blue‐shift in the emission from ≈950 nm to ≈630 nm.^[35]^ Furthermore, we found that 2D perovskites possess superior optoelectronic properties (i.e. longer charge carrier lifetime, higher photoluminescence quantum yield or PLQY). Hence, we reported the first use of such materials (e.g. (PEA)_2_SnI_4_) in visible light LEDs. Since our initial work, research efforts within the scientific community have focused on improving the performance of red 2D Sn HaP LEDs. To this end, a number of strategies have been explored including: i) crystal growth engineering to yield more uniform thin films,[^51^, ^52^, ^53^] ii) dielectric confinement enhancement through organic cation replacement[^54^, ^55^] and iii) the use of antioxidant additives.[^56^, ^57^] To date, (PEA)_2_SnI_4_ remains the champion 2D Sn HaP emitter, with record external quantum efficiencies (EQE) in LEDs of 5 % (Figure 4b)^[57]^ and showing promising upscaling prospects.^[58]^

To attain efficient visible emission beyond red, quantum size effects in 2D HaPs must be combined with I^−^ substitution by smaller halides (e.g. Br^−^), as shown for three‐dimensional (3D) structures. Exploiting this approach, we showed that the emission of (PEA)_2_SnI_4_ can be tuned from ≈630 nm to ≈470 nm by progressively replacing I^−^ by Br^−^ (Figure 4c).^[35]^ This wide emission range makes 2D Sn HaPs interesting candidates for next‐generation LEDs with low toxicity. However, PLQY losses and lower photoluminescence (PL) lifetimes persist as unwanted consequences of Br‐ incorporation,[^35^, ^59^] which make the design of efficient blue Sn HaP LEDs challenging.

### 4.2 Broadband Sn HaP Phosphors

**Figure 5:**
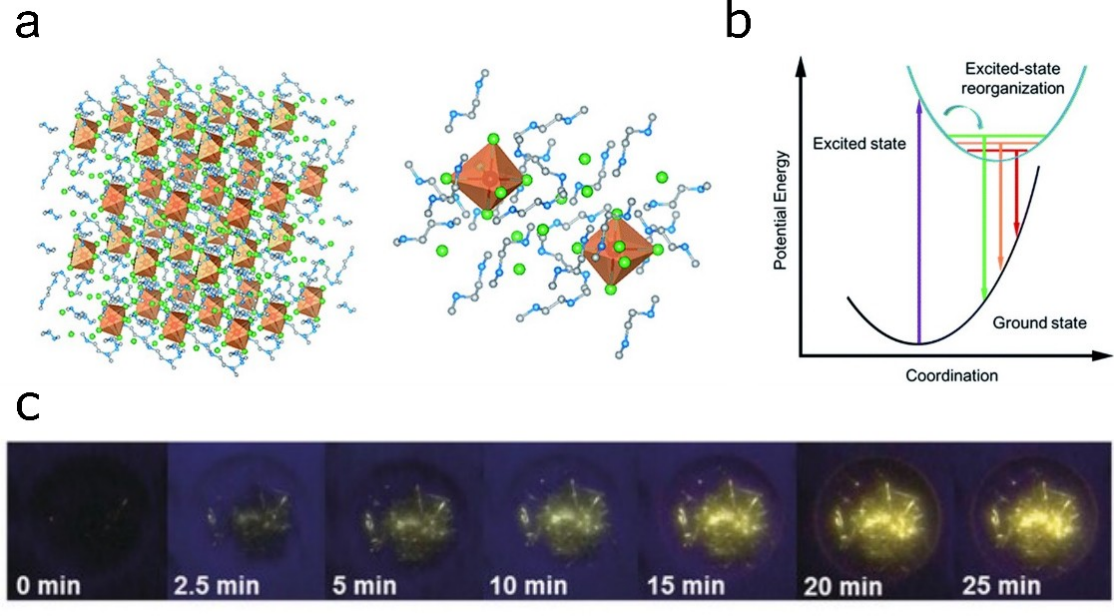
a) Structure of (C_4_N_2_H_14_Br)_4_SnBr_6_ zero‐dimensional phosphor (left) and close‐up view of SnBr_6_^4−^ octahedra isolated by C_4_N_2_H_14_Br^+^ ligands (right); b) process of exciton reorganisation (self‐trapping) and broadband emission. Reproduced with permission from ref. [61]. c) White‐light phosphorescent effect of one‐dimensional C_4_H_14_SnBr_6_ under UV irradiance. Reproduced with permission from ref. [63].

The use of larger surface‐to‐volume ratio systems offers a route to more emissive Br^−^‐based Sn HaPs. For example, Shellaiah et al. demonstrated a PLQY of over 14 % in ligand‐stabilised MASnBr_3_ quantum dots.^[60]^ It is possible that ligand‐mediated surface passivation may mitigate Br^−^‐related losses by reducing energetic disorder. An additional route to boost light emission in Br^−^‐based materials involves the use of strongly confined emitters based on long alkyl chain spacers or even zero‐dimensional hybrid structures (Figure 5a). This has led to extremely long‐lived emission with PLQYs that often approach unity. For example, Zhou et al. achieved PLQYs around 95 % for 0D (C_4_N_2_H_14_Br)_4_SnBr_6_,^[61]^ whereas Zhang et al. reported PLQYs up to 88 % for 2D (C_18_H_35_NH_3_)_2_SnBr_4_.^[62]^ Efficient radiative recombination arises from stronger electron–phonon coupling in the fully isolated inorganic regions, favouring exciton self‐trapping and yielding broadband phosphorescence (Figure 5b,c). Interestingly, this class of Sn HaPs exhibit outstanding stability under a great variety of stressors (i.e. illumination, heat and ambient conditions).^[63]^ The substitution of I^−^ by Br^−^ remains an effective route to increase the stability of low‐dimensional Sn HaP emitters; Zhou et al. demonstrated that (C_4_N_2_H_14_Br)_4_SnBr_3_I_3_ shows higher photostability under illumination vs (C_4_N_2_H_14_I)_4_SnI_6_,^[64]^ following the same rationale as in their 3D counterparts (see above). Overall, these features make Sn HaP phosphors strong candidates for lighting applications beyond monochromatic light such as white‐light phosphorescent devices.

## 5 Outlook: Lessons from Pb HaPs

Unlocking the full potential of Sn HaPs optoelectronics inevitably requires the application of strategies proven useful in their Pb counterparts. This section describes how two popular avenues in Pb HaP solar cells, i.e. defect management and interfacial engineering, can be applied to address issues in Sn HaP devices arising from their halide chemistry.

### 5.1 Halide Defect Management

**Figure 6:**
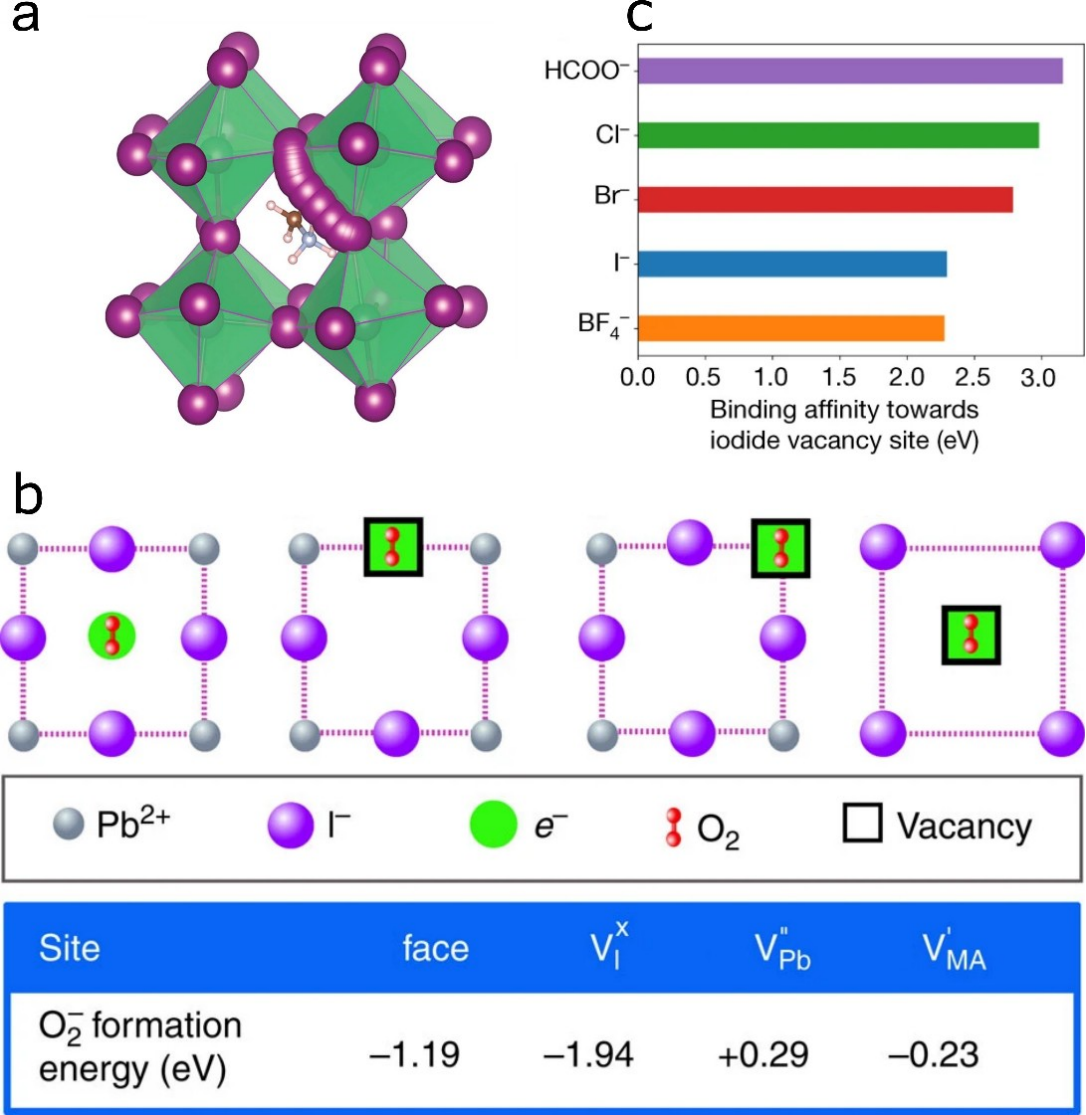
a) I^−^ migration via V_I_ simulated with density functional theory. Reproduced with permission from ref. [65]. b) Possible O_2_^−^ formation sites in MAPbI_3_ and their corresponding formation energies, identifying V_I_ as the most favourable. Reproduced with permission from ref. [69]. c) Calculated binding affinity of pseudohalides to FAPbI_3_ surface. Reproduced with permission from ref. [74].

In the most studied Pb‐based perovskite material, i.e. MAPbI_3_, it is well established that its constituent ions, and especially I^−^, migrate upon external stimuli (e.g. bias or illumination).[^65^, ^66^] Eames et al. identified that V_I_ play a key role in this process by enabling hopping of I^−^ (Figure 6a),^[65]^ while Minns et al. demonstrated that I^−^ can further move through I_i_ positions via the rearrangement of MA^+^ and even form neutral I_2_ within the perovskite lattice.^[67]^ These processes are known to profoundly affect the electronic properties of Pb HaPs by generating trap states and promoting halogen formation. It is reasonable to suppose that such processes can also occur in Sn HaPs. To overcome this, treatments targeting I^−^‐related defects are useful to increase both performance and stability of Pb HaPs; we envisage these strategies to benefit Sn HaPs in a similar manner. Yang et al. introduced additional I^−^ into the organic cation solution to decrease the amount of deep level defects.^[68]^ This route yielded power conversion efficiencies of over 22 % and 19 % for small and large area devices, respectively. Our own approach consisted of applying post‐treatment solutions comprising I^−^ salts onto MAPbI_3_ films to fill V_I_.^[69]^ In our previous work, we demonstrated that upon light and O_2_ exposure V_I_ act as preferred sites for the formation of superoxide (O_2_^−^) species harmful to perovskite (Figure 6b).[^69^, ^70^, ^71^, ^72^] Deactivating O_2_^−^ formation pathways via V_I_ filling also translates into enhanced operational stability in solar cells.^[69]^ While O_2_^−^ formation in Sn HaPs is unlikely due to their prompt reaction with O_2_, passivating halide defects remains important in order to limit potential halogen evolution pathways derived from ion migration.

Caution must be exercised upon treating HaPs with I^−^‐based salts; Tan et al. showed that I_i_ defects may arise from such post‐treatments, promoting unwanted cubic‐to‐hexagonal phase transitions in FAPbI_3_ absorbers^[73]^ and potentially making more I^−^ available for halogen formation in Sn HaPs. As a universal strategy, Jeong et al. recently reported the use of pseudohalide additives for defect elimination in HaP materials.^[74]^ While Br^−^ and Cl^−^ show superior binding affinity to V_I_ vs I^−^, formate (HCOO^−^) pseudohalide provides the strongest interaction in FAPbI_3_ (Figure 6c), leading to i) certified photovoltaic efficiencies of 25.2 %, ii) high operational stability (≈450 h) and iii) bright electroluminescence with EQE over 10 %. While pseudohalides have been implemented in Sn HaPs to control film growth,^[75]^ further additive engineering efforts will continue to enable effective halide defect management strategies.^[76]^ Other methods to mitigate crystallographic defects such as aerosol‐assisted solvent treatments are readily applicable in Pb‐free perovskite technologies.^[77]^

### 5.2 Interfacial Engineering

**Figure 7:**
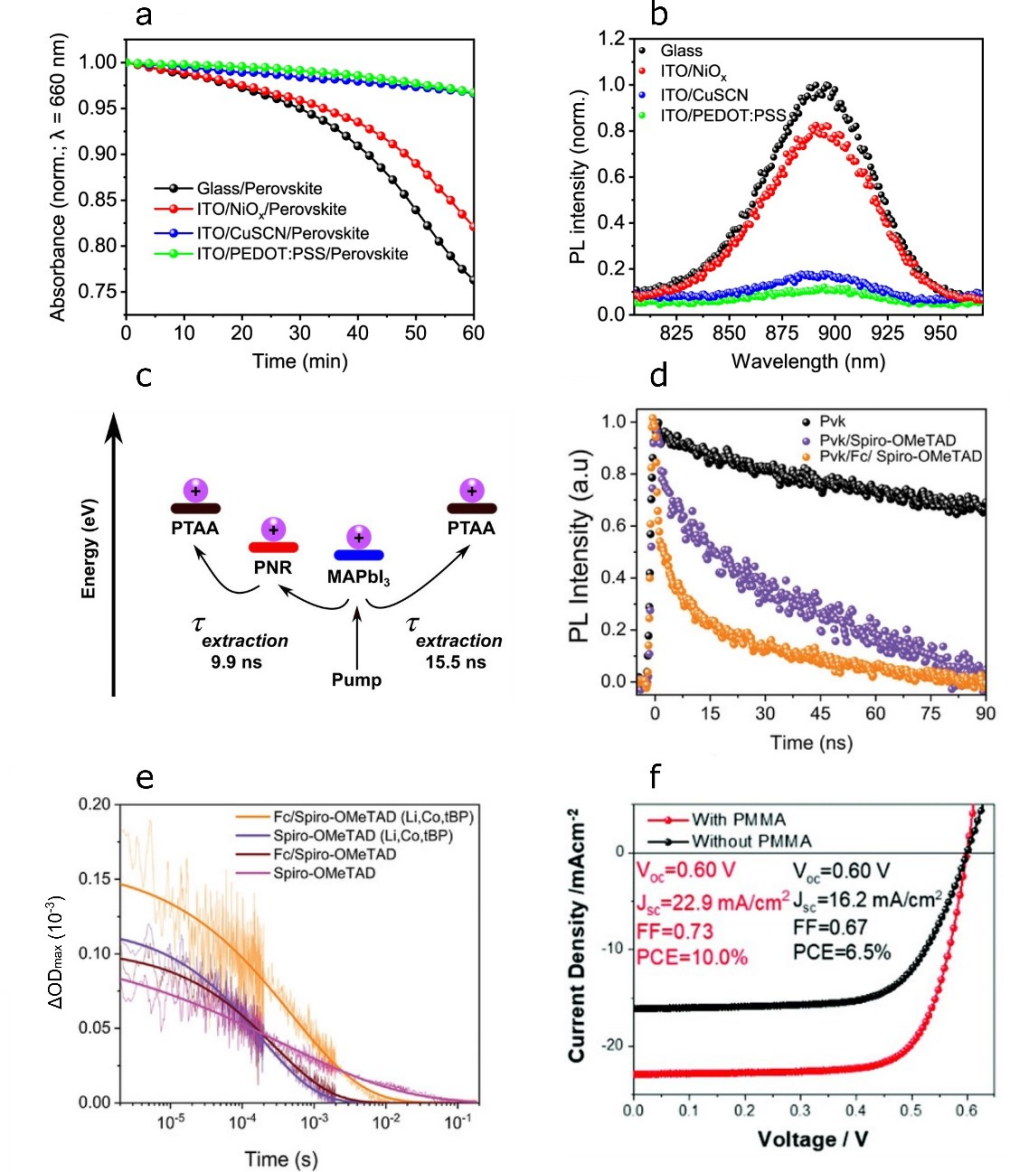
a) Optical degradation test of HTL/(PEA)_0.2_(FA)_0.8_SnI_3_ samples under ambient conditions and b) their quenched PL spectra consistent with hole extraction to HTL. Reproduced with permission from ref. [8]. c) PNRs yield faster hole extraction in PTAA/MAPbI_3_ junctions. Reproduced with permission from ref. [79]. d) Time‐resolved PL decays and e) transient absorption spectroscopy kinetic traces showing enhanced photoinduced charge transfer (i.e. higher hole injection yield) at perovskite/HTL junctions upon ferrocene treatment. Reproduced with permission from ref. [81]. f) Current density–voltage curves of (PEA)_0.2_(FA)_0.8_SnI_3_ solar cells with/without PMMA interlayer engineering. Reproduced with permission from ref. [82].

A wide range of interface engineering strategies have been developed and successfully utilised to improve both the performance and stability of Pb HaP solar cells. For example, regulating Lewis acid–base interactions at HaP/charge transport layer junctions by selecting suitable passivating moieties in the latter is integral to allow efficient charge transfer.^[78]^ However, such approaches remain virtually unexploited in Sn HaPs. We recently observed the ambient stability of (PEA)_0.2_(FA)_0.8_SnI_3_ deposited onto hole transport layers (HTLs) to correlate with the hole extraction ability of the substrate (Figure 7a,b).^[8]^ Here we proposed that the fast removal of holes in perovskite may help to mitigate Sn^2+^‐to‐Sn^4+^ oxidation and the evolution of labile SnI_4_ and enhance stability; this being consistent with the molecular oxygen and moisture‐driven degradation mechanism in Figure 1. Moreover, enhancing anodic contact selectivity in Sn HaP optoelectronics therefore serves as a key device design rule for the realisation of efficient and stable solar cells. A particularly interesting approach involves novel nanomaterials that can extract charges from the device more effectively. Recently, we employed a newly discovered nanomaterial (i.e. phosphorene nanoribbons, PNRs) to improve hole extraction in Pb HaP solar cells (Figure 7c).^[79]^ MAPbI_3_‐based solar cells utilising a PNR/poly(triaryl)amine (PTAA) HTL were shown to exhibit power conversion efficiencies over 21 %, a figure comparable to those in single‐crystalline MAPbI_3_ solar cells.^[80]^ We also exploited ferrocene as a perovskite surface potential modifier at (FAPbI_3_)_0.95_(MAPbBr_3_)_0.05_/*spiro*‐OMeTAD (HTL) junctions.^[81]^ This avenue yielded i) enhanced charge transfer from perovskite to *spiro*‐OMeTAD (Figure 7d,e) and ii) highly efficient solar cells (23.45 %) with high operational stability (70 % performance retention after 1250 h). Similarly, we demonstrated that ultrathin polymethylmethacrylate (PMMA) interlayers can effectively enhance selectivity at PEDOT:PSS/(PEA)_0.2_(FA)_0.8_SnI_3_ interfaces and boost solar cell performance from 6.5 % to 10 % (Figure 7f).^[82]^ While PMMA interlayers are expected to be beneficial to the operational stability of Sn HaP solar cells, further research in this direction is needed. We expect these approaches to boost hole extraction in archetypal Sn HaP device architectures based on PEDOT:PSS and other less commonly employed HTLs such as nickel oxide (NiO_*x*_)^[83]^ or ambipolar tin oxide (SnO_*x*_).^[84]^ Recently, Song et al. demonstrated the use of self‐assembled monolayers (SAMs) on indium tin oxide (ITO) anodes in p‐i‐n, FASnI_3_‐based solar cells.^[85]^ As also shown in Pb and mixed Sn–Pb HaP devices,[^86^, ^87^] we expect chemically tailored SAMs to become next‐generation hole‐selective contacts in Sn HaP optoelectronics.

Though not being the main focus of this Minireview, the importance of interfacial chemistry at Sn HaP/electron transport layer (ETL) junctions should not be disregarded. Considering that I_2_/I_3_^−^ can be found at the surface of Sn HaPs even in freshly made samples,^[21]^ the design of Sn HaP/ETL interfaces tolerant to such degradation products (Figure 1) also warrants future research efforts. For this, the design and application of interlayers that can react with native I_2_ in Sn HaPs is a promising direction. Exploiting the tendency of I_2_ to bind to polymeric materials^[88]^ may serve as an opportunity to scavenge the halogen out of Sn HaPs as it is generated. Kang et al. recently employed a polyvinylpyrrolidone–I_2_ complex to passivate grain‐boundary defects in Pb HaP solar cells;^[89]^ we propose that the I_2_‐capturing ability of polyvinylpyrrolidone can similarly be applied in in situ protective layers for Sn HaPs.

## 6 Conclusions

This Minireview highlights the importance and implications of halide chemistry on the stability, ecological impact and optoelectronic device performance of Sn HaPs. It is now becoming widely accepted that the evolution of native I_2_ in perovskites can trigger the degradation of Sn HaPs. A simple approach to mitigate this process involves the partial substitution of I^−^ by less reactive Br^−^; we envisage this avenue to become widespread for future breakthroughs in solar cells. Although Sn HaPs offer low bioaccumulation, water/soil acidification via hydrolysis of Sn halides remains a major environmental concern. As such, the development of methods to hinder such hydrolysis is urgently required. In this context, two attractive pathways involve the development of i) Sn HaPs with innate resistance to water and/or ii) Sn‐sequestrating agents. Efficient and stable visible‐emitting Sn HaPs lighting technologies can be realised through the combination of halide engineering and low‐dimensional perovskite structures. In particular, strongly‐confined, Br^−^‐based Sn HaPs show great potential as white‐light phosphorescent emitters. Finally, identifying ion motion phenomena in Sn HaPs and implementing lessons from Pb HaP solar cells on halide defect neutralisation (e.g. V_I_ filling) and device interface engineering (e.g. contact selectivity enhancement) may enable strategies to tackle I_2_‐induced degradation and boost efficiency. Unravelling the intricacies of halide chemistry in this class of materials will open up a myriad of possibilities in Sn HaP optoelectronics.

## Conflict of interest

The authors declare no conflict of interest.

## Biographical Information

*Luis Lanzetta is a Postdoctoral Fellow in the KAUST Solar Center at King Abdullah University of Science and Technology. He obtained his PhD in Chemistry at Imperial College London in the group of Prof Saif A. Haque, working on the synthesis and characterisation of lead‐free perovskites for use in photovoltaic and light‐emitting applications. His research focuses on molecular doping approaches for halide perovskite semiconductors and the study of degradative processes in nanostructured hybrid materials for the design of stable energy conversion applications*.

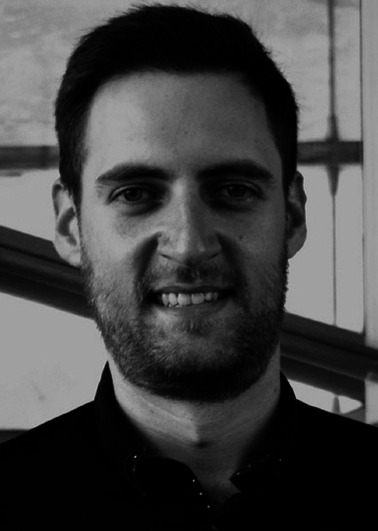

## Biographical Information

*Thomas Webb is a postgraduate researcher in the department of Chemistry at Imperial College London. He holds masters degrees in both Chemistry and Electronics and Electrical Engineering from Imperial College London and the University of Surrey, respectively. He is currently conducting his doctoral studies at Imperial College London investigating the use of novel reductants and developing new strategies to stabilise tin perovskites under the supervision of Prof Saif A. Haque*.

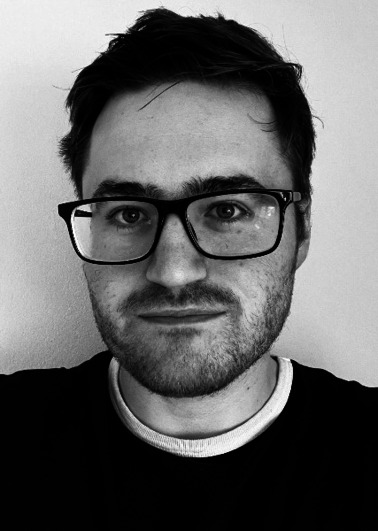

## Biographical Information

*Jose Manuel Marin‐Beloqui is a Maria Zambrano Fellow at the University of Málaga (Spain). His research focuses on the use of transient absorption and Raman spectroscopies to elucidate the excited state dynamics of different materials for use in organic electronics. Previously, he was a postdoctoral researcher at University College London and Imperial College London, analysing charge transfer processes in a wide range of third‐generation photovoltaic devices*.

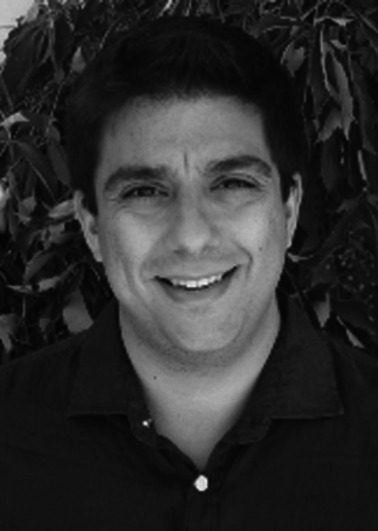

## Biographical Information

*Thomas J. Macdonald is an 1851 Research Fellow in the School of Engineering and Materials Science at Queen Mary University of London. He also holds an Honorary Visiting Researcher position in the Department of Chemistry at Imperial College London. Prior to this, he was a Fellow at Imperial studying the charge carrier dynamics of novel nanomaterials in optoelectronics. From 2017–2019, he was a Ramsay Memorial Fellow at University College London developing new nanomaterials for solar energy conversion. He has a strong interest in renewable energy and extensive experience in the synthesis of functional nanomaterials and the fabrication of third‐generation photovoltaics*.

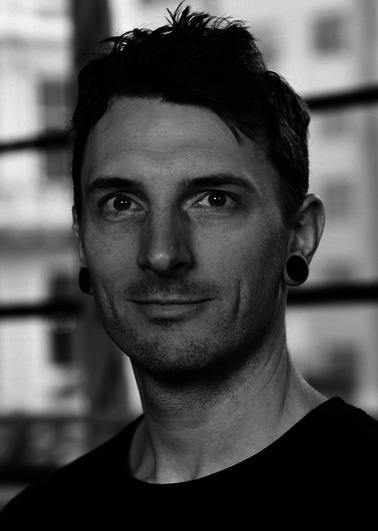

## Biographical Information

*Saif A. Haque is a Professor of Chemistry at Imperial College London. He is a physical chemist with a particular interest in nanomaterials, electronic materials, photochemistry and solar energy conversion. His research is currently addressing the functional characterisation and development of solar cell absorbers and devices based upon solution processable hybrid inorganic–organic semiconducting materials, inorganic metal chalcogenides, quantum dots and perovskites. Prior to becoming a professor, he was a Royal Society University Research Fellow at Imperial between 2005 and 2013*.

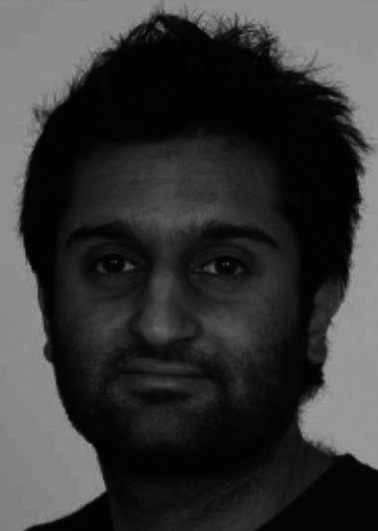

## Supporting information

As a service to our authors and readers, this journal provides supporting information supplied by the authors. Such materials are peer reviewed and may be re‐organized for online delivery, but are not copy‐edited or typeset. Technical support issues arising from supporting information (other than missing files) should be addressed to the authors.

Supporting Information
