# Supplementary material for: Halide Chemistry in Tin Perovskite Optoelectronics: Bottlenecks and Opportunities
Source: Angew Chem Int Ed Engl. 2022 Dec 21;62(8):e202213966. doi: 10.1002/anie.202213966 (PMC10107305; doi:10.1002/anie.202213966)
Supplement: Supplementary file 1 — Supporting Information [file ANIE-62-0-s001.pdf]

Angewandte Chemie
Eine Zeitschrift der Gesellschaft Deutscher Chemiker

Supporting Information

Halide Chemistry in Tin Perovskite Optoelectronics: Bottlenecks and Opportunities

L. Lanzetta, T. Webb, J. M. Marin-Beloqui, T. J. Macdonald, S. A. Haque**

Figure 1

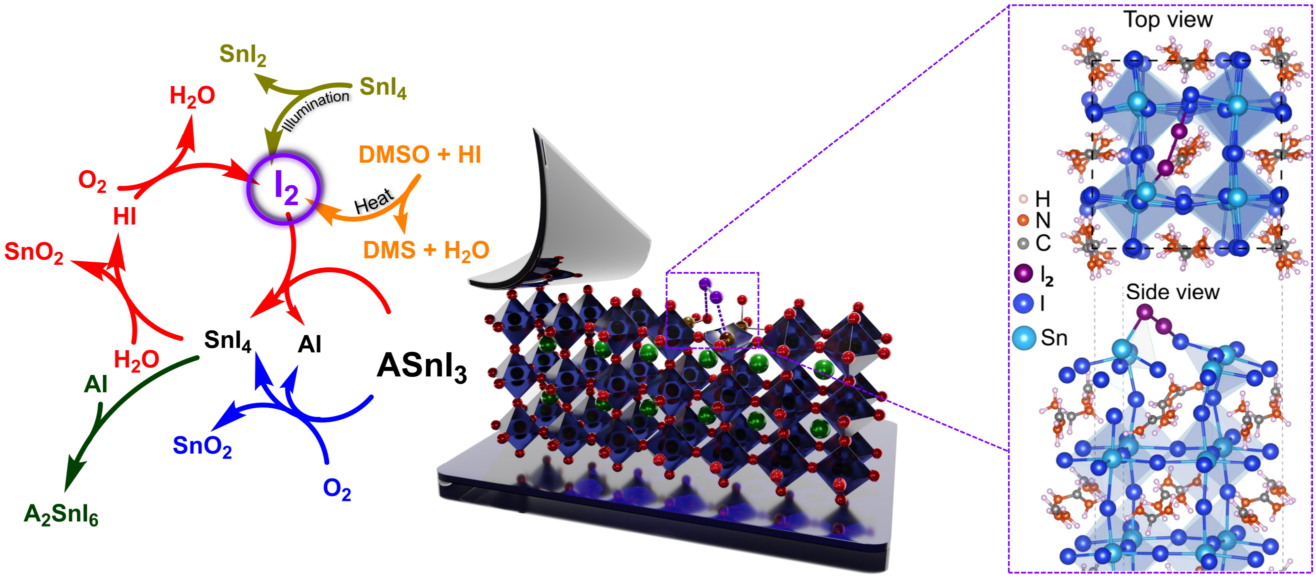

SnI_2
SnI_4
Illumination
H_2O
O_2
I_2
DMSO + HI
Heat
DMS + H_2O
HI
SnO_2
H_2O
SnI_4
AI
$ASnI_3$
AI
SnO_2
O_2
A_2SnI_6
Top view
H
N
C
I_2
I
Sn
Side view

Figure 2

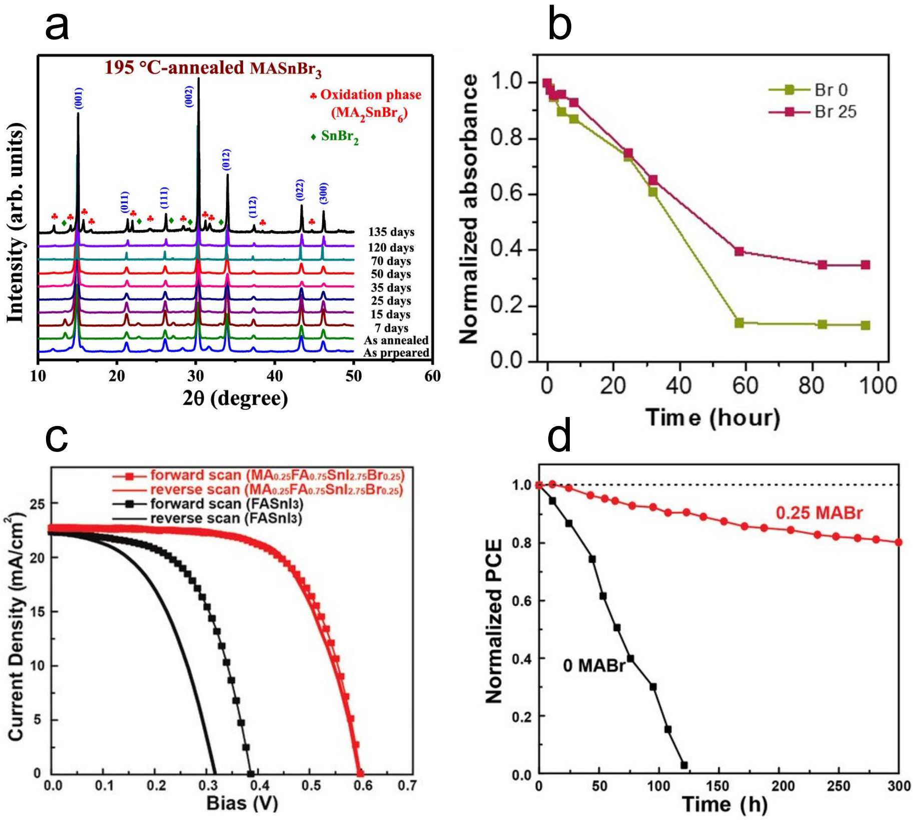
a
195 °C-annealed MASnBr3
Oxidation phase (MA2SnBr6)
SnBr2
(001)
(002)
(011)
(111)
(012)
(112)
(022)
(300)
135 days
120 days
70 days
50 days
35 days
25 days
15 days
7 days
As annealed
As prpeared
Intensity (arb. units)
2θ (degree)
b
Br 0
Br 25
Normalized absorbance
Time (hour)
c
forward scan (MA0.25FA0.75SnI2.75Br0.25)
reverse scan (MA0.25FA0.75SnI2.75Br0.25)
forward scan (FASnI3)
reverse scan (FASnI3)
Current Density (mA/cm2)
Bias (V)
d
0.25 MABr
0 MABr
Normalized PCE
Time (h)

Figure 3

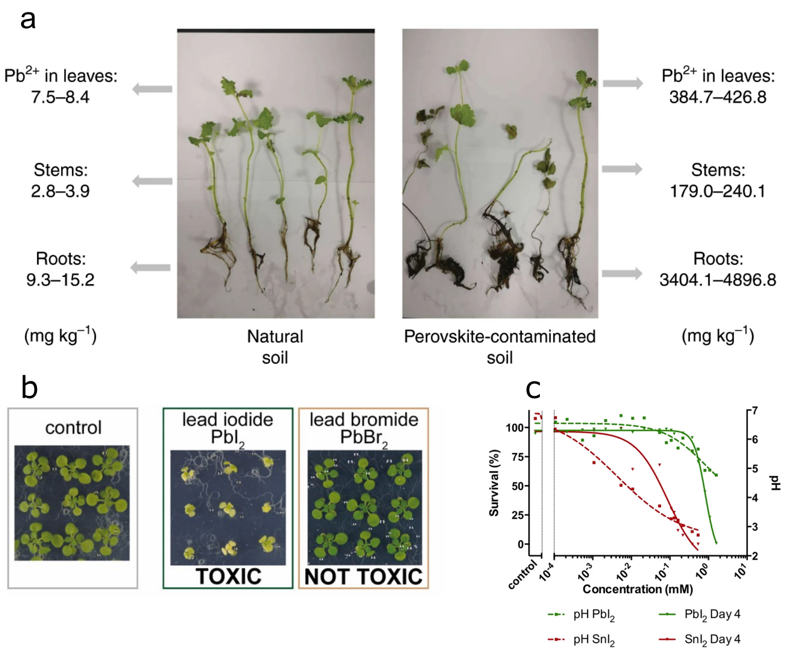

a
Pb2+ in leaves:
7.5–8.4
Stems:
2.8–3.9
Roots:
9.3–15.2
(mg kg−1)
Natural soil
Perovskite-contaminated soil
Pb2+ in leaves:
384.7–426.8
Stems:
179.0–240.1
Roots:
3404.1–4896.8
(mg kg−1)
b
control
lead iodide PbI2
TOXIC
lead bromide PbBr2
NOT TOXIC
c
Survival (%)
pH
Concentration (mM)
control
pH PbI2
PbI2 Day 4
pH SnI2
SnI2 Day 4

Figure 4

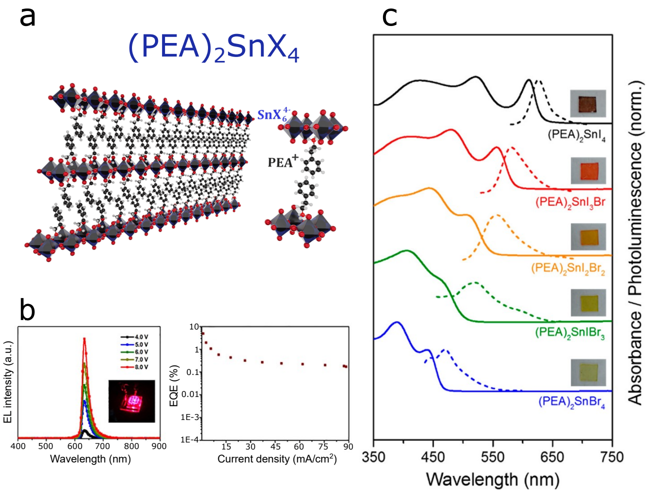
a
(PEA)$_2$SnX$_4$
SnX$_6^{4-}$
PEA$^+$
b
EL intensity (a.u.)
Wavelength (nm)
EQE (%)
Current density (mA/cm^2)
c
(PEA)$_2$SnI$_4$
(PEA)$_2$SnI$_3$Br
(PEA)$_2$SnI$_2$Br$_2$
(PEA)$_2$SnIBr$_3$
(PEA)$_2$SnBr$_4$
Absorbance / Photoluminescence (norm.)
Wavelength (nm)

Figure 5

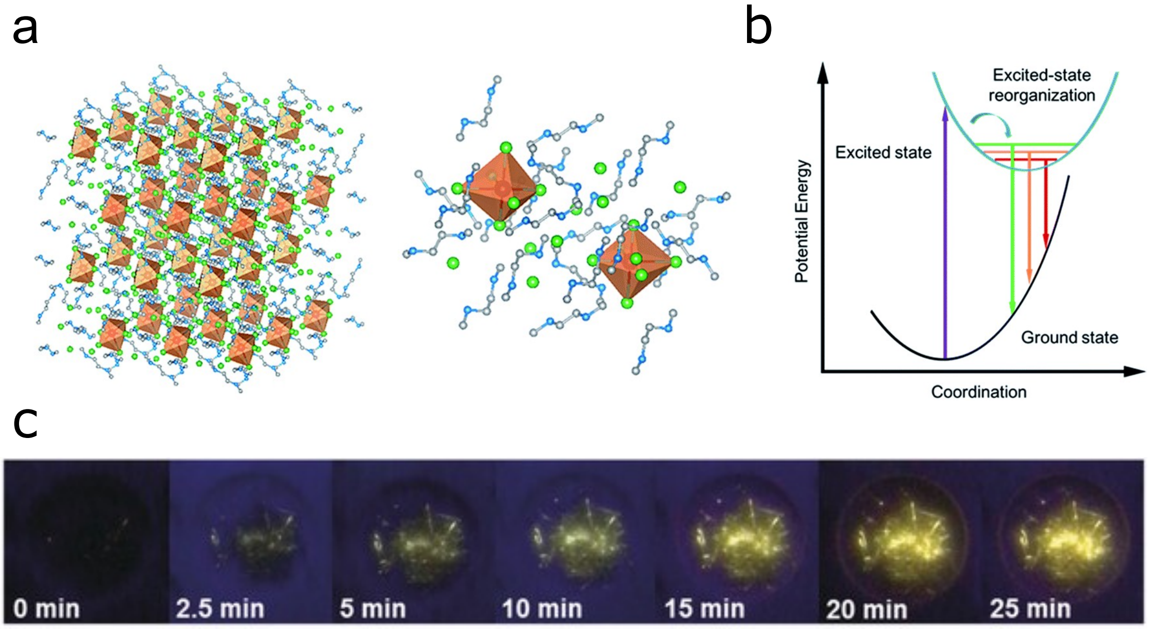
a
b
Excited-state
reorganization
Excited state
Potential Energy
Ground state
Coordination
c
0 min
2.5 min
5 min
10 min
15 min
20 min
25 min

Figure 6

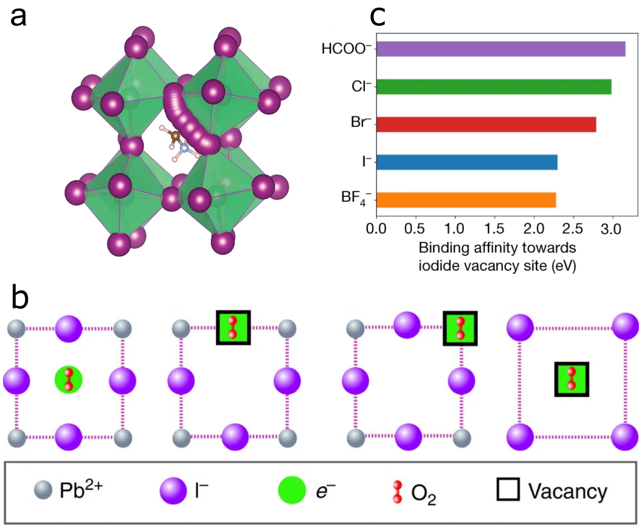

Site	face	V_I^x	V_{Pb}''	V_{MA}'
O_2^- formation energy (eV)	–1.19	–1.94	+0.29	–0.23

Figure 7

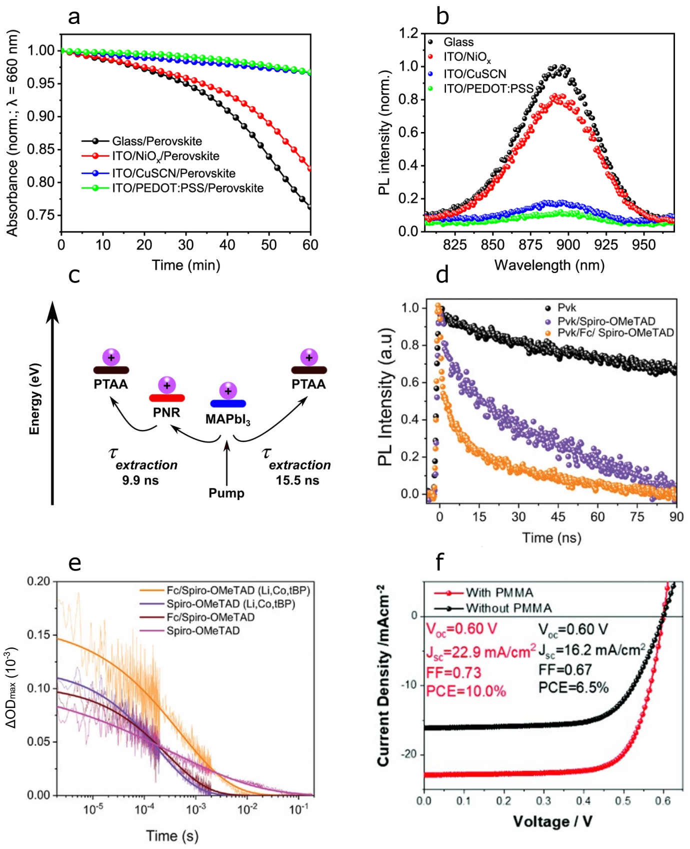
a
Absorbance (norm.; λ = 660 nm)
Glass/Perovskite
ITO/NiOx/Perovskite
ITO/CuSCN/Perovskite
ITO/PEDOT:PSS/Perovskite
Time (min)
b
PL intensity (norm.)
Glass
ITO/NiOx
ITO/CuSCN
ITO/PEDOT:PSS
Wavelength (nm)
c
Energy (eV)
PTAA
PNR
MAPbI3
PTAA
τ extraction 9.9 ns
τ extraction 15.5 ns
Pump
d
PL Intensity (a.u)
Pvk
Pvk/Spiro-OMeTAD
Pvk/Fc/ Spiro-OMeTAD
Time (ns)
e
ΔODmax (10⁻³)
Fc/Spiro-OMeTAD (Li,Co,tBP)
Spiro-OMeTAD (Li,Co,tBP)
Fc/Spiro-OMeTAD
Spiro-OMeTAD
Time (s)
f
Current Density /mAcm⁻²
With PMMA
Without PMMA
Voc=0.60 V
Jsc=22.9 mA/cm²
FF=0.73
PCE=10.0%
Voc=0.60 V
Jsc=16.2 mA/cm²
FF=0.67
PCE=6.5%
Voltage / V

Frontispiece

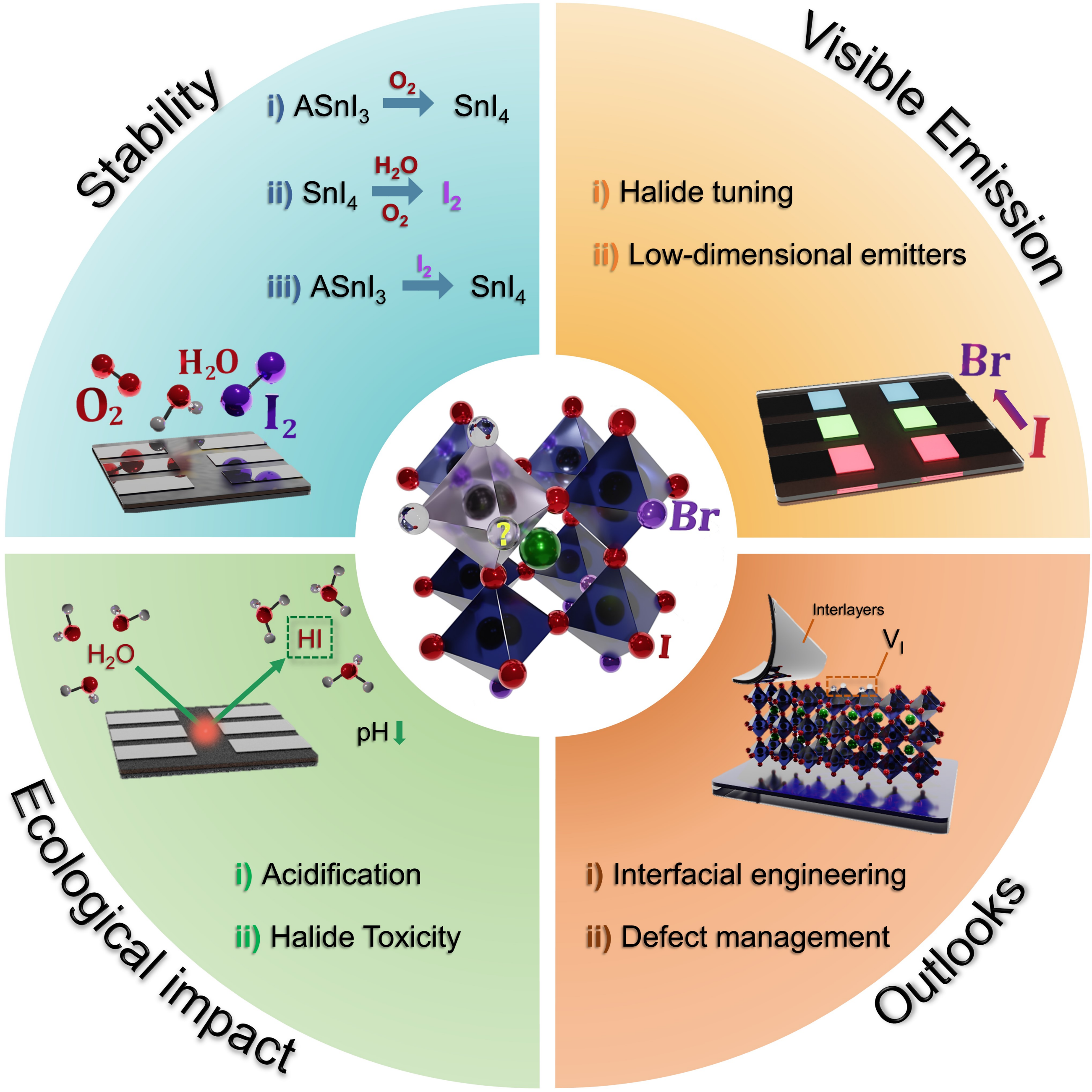

Table of Contents

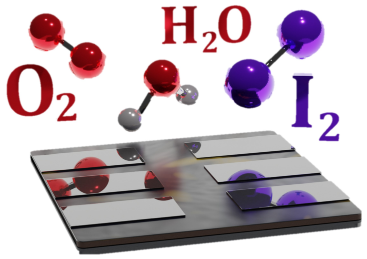

Portraits

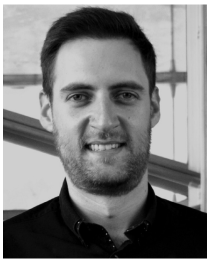

Luis Lanzetta

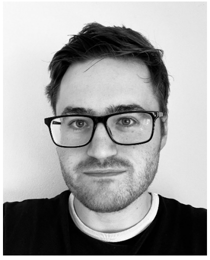

Thomas Webb

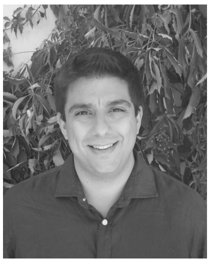

Jose Manuel Marin-Beloqui

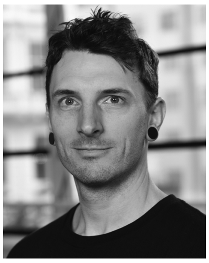

Thomas J. Macdonald

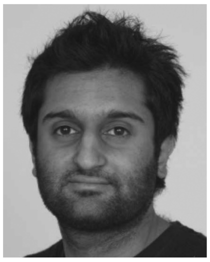

Saif A. Haque
